# Genome-wide identification and characterization of DNA enhancers with a stacked multivariate fusion framework

**DOI:** 10.1371/journal.pcbi.1010779

**Published:** 2022-12-15

**Authors:** Yansong Wang, Zilong Hou, Yuning Yang, Ka-chun Wong, Xiangtao Li

**Affiliations:** 1 School of Artificial Intelligence, Jilin University, Changchun, China; 2 Donnelly Centre for Cellular and Biomolecular Research, University of Toronto, Toronto, Canada; 3 Department of Computer science, City University of Hong Kong, Hong Kong, Special Administrative Region; University of Illinois at Urbana-Champaign, UNITED STATES

## Abstract

Enhancers are short non-coding DNA sequences outside of the target promoter regions that can be bound by specific proteins to increase a gene’s transcriptional activity, which has a crucial role in the spatiotemporal and quantitative regulation of gene expression. However, enhancers do not have a specific sequence motifs or structures, and their scattered distribution in the genome makes the identification of enhancers from human cell lines particularly challenging. Here we present a novel, stacked multivariate fusion framework called SMFM, which enables a comprehensive identification and analysis of enhancers from regulatory DNA sequences as well as their interpretation. Specifically, to characterize the hierarchical relationships of enhancer sequences, multi-source biological information and dynamic semantic information are fused to represent regulatory DNA enhancer sequences. Then, we implement a deep learning–based sequence network to learn the feature representation of the enhancer sequences comprehensively and to extract the implicit relationships in the dynamic semantic information. Ultimately, an ensemble machine learning classifier is trained based on the refined multi-source features and dynamic implicit relations obtained from the deep learning-based sequence network. Benchmarking experiments demonstrated that SMFM significantly outperforms other existing methods using several evaluation metrics. In addition, an independent test set was used to validate the generalization performance of SMFM by comparing it to other state-of-the-art enhancer identification methods. Moreover, we performed motif analysis based on the contribution scores of different bases of enhancer sequences to the final identification results. Besides, we conducted interpretability analysis of the identified enhancer sequences based on attention weights of EnhancerBERT, a fine-tuned BERT model that provides new insights into exploring the gene semantic information likely to underlie the discovered enhancers in an interpretable manner. Finally, in a human placenta study with 4,562 active distal gene regulatory enhancers, SMFM successfully exposed tissue-related placental development and the differential mechanism, demonstrating the generalizability and stability of our proposed framework.

## Author summary

Numerous evidence suggest that genes regulated by enhancers located in non-coding DNA regions are involved in a myriad of biological activities. To fully understand the regulatory role and mechanisms of enhancers on genes, the localization and identification of enhancers is essential. Several experimental biological methods are capable of localizing enhancers, however, these methods are resource intensive. To address this limitation, we developed a stacked multivariate fusion framework, called SMFM to identify and analyze enhancers with high accuracy and efficiency based on enhancer-specific dynamic semantic information and multi-source biological properties. The performance of the model is verified by experiments comparing different feature algorithms and classification algorithms. The superiority of our method is demonstrated by comparing it with several state-of-the-art algorithms. In addition, several analytical experiments demonstrate that SMFM is capable of recognizing enhancers in different tissues and detecting motifs in enhancers. To the best of our knowledge, this is the first computational approach that uses enhancer-specific dynamic semantic information to identify enhancers from regulatory DNA sequences and interpret them. It is expected that the SMFM model will effectively target enhancers and provide valid candidates for further biological experiments.

This is a *PLOS Computational Biology* Methods paper.

## Introduction

Enhancers are a series of DNA segments in the non-coding DNA sequences that can significantly increase the transcription rate of their target genes after being bound by transcriptional factors and other co-regulators that control the promoters of the associated genes [1]. Recent studies have shown that different enhancers have distinct sets of subregions (or motifs) that bind specific transcription factors, and exhibit diverse activities and regulatory roles on multiple biological genes [2]. Enhancers are typically in the intergenic and intronic regions and often include binding sites for multiple transcription factors. Intriguingly, active enhancers undergo transcription by RNA polymerase II to generate enhancer RNAs (eRNAs) [3, 4]. Moreover, genetic variants in cell-type-specific enhancer sequences are associated with a risk for common diseases in humans [5]. Therefore, it is of great interest to identify enhancers in regulatory DNA sequences with the potential to provide new opportunities for understanding physiological and pathological processes.

In the early days, researchers identified enhancers primarily by conducting biological experiments with vitro and vivo functional assays, such as gel-shift assays in [6]. More recent approaches use publicly available comparative sequence datasets for comparative genomics [7], for example. However, the heavy cost and tedious processing times of high-throughput experiments severely restrict their practical application [8]for effective enhancer identification, due to the lack of sample diversity [7] and the difficulty in simulating different cellular conditions [9]. Currently, a series of computational methods have been developed to address enhancer identification, which can be divided into three categories: *1) Chromatin-based methods*: these algorithms typically employ chromatin information to characterize enhancer sequences, and then most identify enhancers using various machine learning classifiers, including ChromaGenSVM [10], RFECS [11], EnhancerFinder [12], GKM-SVM [13]. *2) Physicochemical-based methods*: such algorithms are implemented using various physicochemical features that encode enhancer subsequences, including iEnhancer-2L [14], EnhancerPred [15], iEnhancer-EL [16], iEnhancer-RF [17], iEnhancer-XG [18], iEnhancer-ECNN [19], CSI-ANN [20] and Enhancer-IF [21], where iEnhancer-ECNN [19] and CSI-ANN [20] utilize deep learning techniques to learn the implicit information in the features, and the other methods use traditional machine learning classifiers to accomplish the identification task. *3) Contextual-based methods*:iEnhancer-EBLSTM [22], iEnhancer-5Step [23] and BERT-2DCNNs [24] consider the contextual information in enhancer sequences, and use different natural language processing technologies to form the embedding matrix of enhancer sequences. However, most of these computational models use only a single feature type to characterize enhancer sequences, making it difficult to describe distribution and the representations between nucleotides and their contexts, leaving adequate room for improving performance.

In our study, we designed a novel stacked multivariate fusion model, called SMFM. In SMFM, multi-source biological features and EnhancerBERT are proposed to represent the enhancer sequences, where EnhancerBERT can maximize the characterization power of the dynamic semantic information of enhancer sequences. Then, we designed a deep learning-based sequence network to learn the dynamic implicit relations and long-distance dependencies in the dynamic semantic information. Finally, we merged the two types of processed features and feed them into an ensemble machine learning classifier to derive the final prediction results. To validate the effectiveness and good performance of SMFM, we conducted several experiments performing a rigorous 10-fold cross-validation on the training set. The experimental results showed that SMFM significantly outperforms currently available methods. In addition, to verify the stability and generalization ability of SMFM, we tested and compared and compared the conduct of SMFM on a completely independent test set and results indicated that SMFM generally outperforms existing methods. Furthermore, to explore the ability of characterization of SMFM for tissue-specific enhancers, we designed a stepwise experiment on 4,562 placental enhancers: identifying placental enhancers in the first step and distinguishing placental enhancers from enhancers in other tissues in the second step. In order to validate the effectiveness of placental enhancers identified by SMFM, we then performed gene ontology (GO) and kyoto encyclopedia of genes and genomes (KEGG) enrichment analysis based on results of stepwise experiments. Finally, we carried out motif analysis and interpretability analysis of the identified enhancer sequences based on attention weights in EnhancerBERT and provide here an online web server that can predict enhancers in DNA sequences online, which is available at http://39.104.69.176:5010/.

## Materials and methods

### A. Data sources

We collected the dataset from nine different cell lines, including H1ES, K562, GM12878, HepG2, HUVEC, HSMM, NHLF, NHEK and HMEC [14]. The samples in the dataset were selected based on chromatin state information, which was annotated by ChromHMM [25], and divided into 200bp fragments to match linker and nucleosome length DNA. A sample was discarded if its length was less than 200bp. The CD-HIT tool was utilized for reducing the similarity between fragments with a threshold value of 0.8. From this, we obtained the dataset including three classes: strong enhancers (${S^{+}}_{strong}$), weak enhancers (${S^{+}}_{weak}$) and non-enhancers ($S^{-}$). In our work, we merged the ${S^{+}}_{strong}$ and ${S^{+}}_{weak}$ as the positive samples ($S^{+}$), while $S^{-}$ were the negative samples following reference [14]. The structure of the dataset can be described as follows:

$$\left\{ \begin{array}{l} S=S^{+}\cup S^{-}\\ S^{+}=S^{+}{}_{strong}\cup S^{+}{}_{weak} \end{array}\right.$$

The dataset includes 2968 samples, of which 1484 are enhancers and the others non-enhancers. We evaluated the performance using 10-fold cross-validation, which divides the training set into 10 subsets, where one subset is the validation set, and the other 9 subsets constitute the training set. Each subset needs to be performed once as a validation set. In addition, we employed an independent test set including 200 enhancers and 200 non-enhancers to test the stability and generalization ability of SMFM compared with other existing methods.

**Fig 1:**
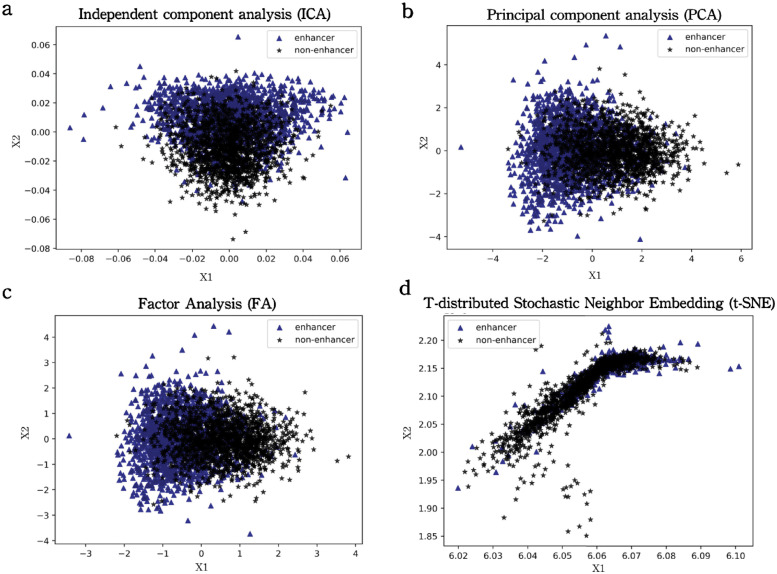
Dataset visualization for DNA Enhancers based on the one-hot encoding approach. All figures are drawn using dimensionality reduction methods including ICA, PCA, FA and t-SNE with Python scikit-learn package as default setting. From the figures it can be concluded that linear classification cannot be utilized in enhancer dataset.

To visualize the enhancer dataset, we applied a series of dimensionality reduction methods to project the sequence feature representation based on the one-hot encoding approach to the two-dimensional space, as envisioned in Fig 1. Unfortunately, it can be observed that the enhancer dataset cannot be classified linearly. Therefore, the development of effective sequence representation models and nonlinear-based modeling including deep neural networks is imperative to identify these sequences in human cell lines.

### B. Feature representation schemes

To characterize enhancer sequences as efficiently as possible, two types of quantifiable features including multi-source biological information and dynamic semantic information are usually adopted in research to represent regulatory DNA enhancer sequences.

*1) Positional gapped k-m-tuple pairs (PGKM)*: To capture interactions between non-adjacent residues, gapped k-mer feature generation method is often employed to represent the enhancer sequence for the classification tasks [26]. However, such a method discards information about the positions of the different functional subsequences (motifs), which have an important role in recording the distinction between the particular functional sequences (e.g. enhancers). To overcome this limitation, we introduce the positional gap k-m-tuple pair (PGKM) as one of the feature descriptors. PGKM contains three parts: k-tuple ({*Nu* × *k*}), m-tuple ({*Nu* × *m*}), and gap (*G*). The feature generation procedure can be characterized as follows:

PGKM={Nu×k},(Gap),{Nu×m},

where *Nu* ∈ {*A*, *C*, *G*, *T*}, *Gap* represents the number of nucleotide intervals between tuples, for *Gap* = *n*, PGKM will calculate the nucleotides between two tuples less than or equal to n, with a lower bound of 1. *k* denotes the number of nucleotides in the first tuple, and *m* denotes the number of nucleotides in the second tuple, respectively. Therefore, in general, when *Gap* = *n*, PGKM can generate 4^*k*^ × 4^*m*^ × *n* features for an enhancer sequence.

Considering the sequence ‘ACCGTA’ as an example, PGKM counts the number of times each tuple pair appears in the sequence and uses this number as the value of the corresponding feature, when *Gap* = 3, *k* = 1, *m* = 1, 48 tuple-pairs (features) can be generated, including three cases: 1) when *Gap* = 1, the following features are calculated: A_A, A_C, A_G, A_T, C_A, C_C, C_G, C_T, G_A, G_C, G_G, G_T, T_A, T_C, T_G, T_T; 2) when *Gap* = 2, A_ _A, A_ _C, A_ _G, A_ _T, C_ _A, C_ _C, C_ _G, C_ _T, G_ _A, G_ _C, G_ _G, G_ _T, T_ _A, T_ _C, T_ _G, T_ _T are calculated; 3) when *Gap* = 3, there are 16 features as follows: A_ _ _A, A_ _ _C, A_ _ _G, A_ _ _T, C_ _ _A, C_ _ _C, C_ _ _G, C_ _ _T, G_ _ _A, G_ _ _C, G_ _ _G, G_ _ _T, T_ _ _A, T_ _ _C, T_ _ _G and T_ _ _T. On this basis, the given sequence has: ∑*A*_*C* = 1, ∑*C*_*G* = 1, ∑*C*_*T* = 1, ∑*G*_*A* = 1, ∑*A*_ _*G* = 1, ∑*C*_ _*T* = 1, ∑*C*_ _*A* = 1, ∑*A*_ _ _*T* = 1, ∑*C*_ _ _*A* = 1. In addition, the value is set to 0 for the remaining features as they do not appear in the sequence ‘ACCGTA’.

*2) Pseudo K-tuple nucleotide composition (PseKNC)*: To extract local contextual features from the enhancer sequences, PseKNC is employed to encode the nucleotide sequences, which can embrace the adjacent information of each nucleotide in the sequences [27]. Specifically, the regular k-tuple is a vector that represents a nucleotide sequence with size of 4^*k*^. The PseKNC can be applied by aggregating the set of k-tuples that contains all tuples consisting of less than or equal to *k* nucleotides. It can be defined as follows:

$$V_{i}=\left[f_{1}^{i-tuple}\;,\;f_{2}^{i-tuple}\;,\;f_{3}^{i-tuple}\;,\;...\;,\;f_{t}^{i-tuple}\;,\;...\;,\;f_{4^{i}}^{i-tuple}\;\right]\;\left(1\leq i\leq k\right),$$

where *V*_*i*_ represents the vector generated by i-tuple and $f_{t}^{i}$ denotes the frequency of t-th i-tuple in a sequence. We set k = 3, which yields vectors corresponding to mononucleotide tuples, dinucleotide tuples and trinucleotide tuples. On this basis, each enhancer sequence would be depicted as a one-dimensional vector with size *V*_1_ + *V*_2_ + *V*_3_.

*3) Nucleotide physicochemical properties (NPCP)*: Apart from the nucleotide distribution representation, the physicochemical property is a fundamental property of a nucleotide that provides a unique contribution to characterize the sequences. Here four different physicochemical properties including Zcurve [28], GC-content [29], (A+T)/(C+G) ratio [30], and GC/AT skew [31], are employed to represent the enhancer sequence, which can generate 3-, 1-, 1- and 2-dimensional vectors, respectively. Therefore, the NPCP for the *t*th sequence *s*_*t*_ can be formulated as follows:

$$NPCP\left(s_{t}\right)=concatenate\left(f_{i}\left(s_{t}\right)\right)\;\left(1\leq i\leq 4\right),$$

where *f*_*i*_ indicates the *i*-th property in NPCP.

*4) Multi-source feature selection*: Since multi-source biological information yields excessive features, this leads to a very laborious training process of the model and also prevents the model from capturing the most critical information that distinguishes the different enhancer subsequences. To address these limitations, we propose employing an AdaBoost model to identify the best subset of features from these high-dimensional features. Specifically, the selector in the AdaBoost model scores the different features by partitioning each feature into all the trees trained on instances with different weight distributions, and calculating the average impurity reduction for each feature. After obtaining the scores of all features, the 472 refined features with an average impurity curtailment over zero are selected as the final streamlined feature set.

*5) Enhancer dynamic semantic information (EnhancerBERT)*: BERT (bidirectional encoder representations from transformers) can learn powerful representations of language to encode information about syntax and semantics, and which is typically pre-trained on a large corpus in a self-supervised fashion [32]. In this context, it is natural to consider enhancer sequences as texts and to explore the semantic information between them by considering nucleic acids as words in a biological language, and structural and regulatory functions as syntactic and semantic information in the enhancer sequence. Inspired by reference [33], we developed EnhancerBERT to maximize the characterization power of the dynamic semantic information of enhancer sequences. In our EnhancerBERT model, we tokenize the enhancer sequences to make them more syntactic, while the prediction task of the BERT-based model shifts to make predictions on how many continuous tokens in an enhancer ‘sentence’ match the possible realistic cases. Indeed, considering that the use of a single acid as a token is too rare, we use *k*-mer (*k* is an integer greater than zero) to process the enhancer sequences. For the sequence ‘ATCGGGCTA’, when *k* = 3, the tokens {ATC, TCG, CGG, GGG, GGC, GCT, CTA} will be generated after 3-mer processing. Note that we have added two special tokens: [CLS] to represent the beginning of the sequence and [SEP] to represent the end of the sequence following reference [33]. Therefore, 4^*k*^ + 2 tokens can be obtained in the vocabulary of kmer. After that, the EnhancerBERT model is pre-trained on a set of masked enhancer sequences that are processed as a series of *k*-mer tokens, each of which can be represented as a unique numerical vector. That is, each sequence can be represented as a matrix *M*. On this basis, EnhancerBERT captures contextual information using a multi-headed self-focus mechanism on *M*, which is described as follows:

$$MultiHead\left(Q,K,V\right)=Concatenate\left(head_{1},head_{2},...,head_{n}\right)W^{O}$$

$$head_{i}=Attention\left(Q,K,V\right)$$

$$Attention\left(Q,K,V\right)=softmax(\frac{QK^{T}}{\sqrt{d_{k}}})\cdot V,$$

where

$$\left\{ \begin{array}{l} Q=M\cdot W_{i}^{Q}\\ K=M\cdot W_{i}^{K}\\ V=M\cdot W_{i}^{V}, \end{array}\right.$$

*Q*, *K*, *V* represents query, key and value respectively, which are projected by *n* diverse linear conversions. ${\left\{W_{i}^{Q},W_{i}^{K},W_{i}^{V}\right\}}_{i=0}^{n}$ are the learnable parameter matrices of the linear projection, respectively. Each *head*_*i*_ is utilized to compute the next hidden state of the matrix *M*, first calculating the attention fraction between every two tokens and then appending rows in $MW_{i}^{V}$ using them as weights. After that, *MultiHead* concatenates *head*_1∼*n*_ with a distinct set of $\left\{W_{i}^{Q},\;W_{i}^{K},\;W_{i}^{V}\right\}$. The whole process is conducted *T* times and *T* is the number of layers.

In the process of fine-tuning the model, we remove the head of the pre-trained model and replace it with a random initialization. Regarding the hyperparameters used for fine-tuning, we fine-tune EnhancerBERT for five epochs on the enhancer training set and apply an early stopping mechanism with a patience of two to prevent overfitting phenomena. A roll back mechanism of the model parameters is utilized after an early stop mechanism and the Adam, without weight decay, is chosen as the optimizer. Remarkably, the aforementioned hyperparameters are consistent for all EnhancerBERT (including 4 models, from 3 to 6mers, respectively), which also use 12 Transformer encoder layers, each consisting of 12 self-attentive heads, to extract semantic information using a multi-headed self-attentive mechanism. Moreover, in our study, to capture sufficient multilayer fusion enhancer information, we simply extract the hidden states from the last layer of the model and drop the vector representation obtained from the special tokens [CLS] and [SEP] added before and after each enhancer sequence to generate the (200-*k*+1, 768) matrix, where *k* is the value of kmer used to process the enhancer sequences, and 768 is the dimension of the vector generated by EnhancerBERT for each token.

### C. Stacked multivariate fusion model (SMFM)

**Fig 2:**
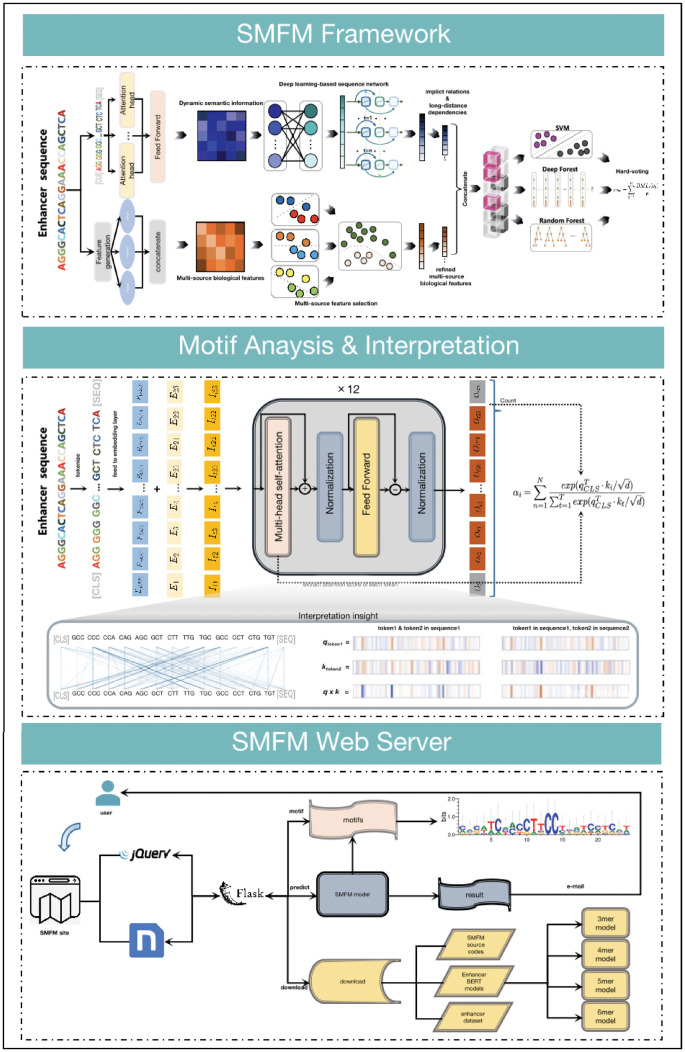
(a) The overall framework of SMFM. First, enhancer sequences are generated as multi-source biological features and dynamic semantic information utilizing multi-source feature generation and EnhancerBERT, which are then fed into a multi-source feature selector and deep learning-based sequence network, respectively. Finally, the streamlined information is combined as input for the ensemble machine learning classifier to produce the final prediction results. (b) The motif analysis for EnhancerBERT and corresponding interpretation. We extract the attention heads of EnhancerBERT to calculate attention scores of each token, and motifs are found by using filter conditions of attention scores. Corresponding interpretations are performed to analyse attention process of EnhancerBERT and the regions that EnhancerBERT concentrated on. (c) The workflow and function display of the SMFM web server. The web server has three functions: predicting enhancer sequences, motif analysis pipeline and downloading EnhancerBERT models and source codes of SMFM.

To capture efficiently the information contained in multiple feature scenarios that are critical for enhancer characterization, we designed a novel stacked multivariate fusion model, called SMFM including three important components, as shown in Fig 2. As depicted in this figure, rather than traditional machine learning or deep learning approaches, SMFM synergizes the two in a stacked fashion. First, the dynamic semantic information obtained by EnahncerBERT is directly fed into the deep learning-based sequence network to learn the implicit semantic information and long-range dependencies. Then, refined features are obtained by scoring multi-source biological information using a multi-source feature selection model. Afterwards, based on the integration of the both features mentioned above, we propose an ensemble machine learning classifier to predict enhancers in human cell lines, where SVM [34], Deep Forest [35] and Random Forest [36] are adopted as the individual classifiers of the ensemble model.

*1) Deep learning-based sequence network*: In this section, SMFM first feeds the dynamic semantic vectors into the one dimensional convolutional neural network (1D CNN) to learn the implicit relationships in the enhancer sequences as it has previously shown potential and significance in relation to the local feature extraction and sequence data prediction [37]. Then, each layer of SMFM performs a linear transformation of the output of the previous layer by multiplying by a weight matrix. Indeed, each filter in a kernel has different weight parameter matrices, *M*, as well as bias vectors *b*. For each convolution kernel, it scans the original semantic vectors *R*_*k*_ with stride size and does matrix multiplication on the scanned area of features according to the perceptual field, then it superimposes the results of the above operations to obtain the bias vector. Mathematically, a convolutional layer is computed as follows:

$$vector\left(x,y\right)=ReLU(\sum_{k=1}^{n}\left(R_{k}\times M_{k}\right)\cdot \left(x,y\right)+b),$$

where *n* is the number of matrices obtained from the convolution kernel, each *vector* calculated by the above equation characterizes the value of the element at the corresponding position in the matrix *M*. In addition, *ReLU* is an activation function that enables the network to learn complex forms in input data, which can be defined as follows:

$$ReLU\left(x\right)=\{\begin{array}{l} x,\;if\;x\geq 0\\ 0,\;if\;x<0. \end{array}$$

From these, the output of the two layers of the deep convolution network is enriched with implicit semantic relations, which are significant for the representation of the enhancers.

Simultaneously, to address the long-distance dependencies available in the enhancer sequences, SMFM uses a Bidirectional long-short-term memory network that includes a conditional random field layer in conjunction with attention-based feature modeling to identify DNA enhancers in human cell lines. Compared to traditional recurrent neural networks (RNNs), our model is advantageous in resolving gradient disappearance or explosion, while allowing capturing long-term dependencies. Intuitively, the implicit semantic vectors are presented forwards and backwards in two separate networks available for the enhancer sequences and then connected to the same output layer. The forward LSTM reads an input implicit semantic vector from beginning to end and the backward LSTM reads the same input vector from back to front. Specifically, for the *t*-th time step, the current forgetting factor (*f*_*t*_) can be calculated using the hidden state of the last time step *H*_*t*−1_ and the implicit semantic vector learned from the enhancer sequences of the current time step *I*_*t*_:

$$f_{t}=\sigma (W_{f}\cdot \left[H_{t-1},\;I_{t}\right]+b_{f})\;,$$

where *σ* is the logistic sigmoid function, and *W*_*f*_ is a trainable weight of the forget gate in BiLSTM. After that, the model regulates the percentage of the implicit semantic vector *I*_*t*_ flowing into the memory cell by using two functional modules. One module controls the inflow percentage by generating a control signal *s*_*t*_, and the other module calculates the candidate memory cell $M_{t}^{\prime }$ based on the *tanh* layer and *s*_*t*_.

$$s_{t}=\sigma (W_{i}\cdot \left[H_{t-1},\;I_{t}\right]+b_{s})$$

$$M_{t}^{\prime }=tanh(W_{M}\cdot \left[H_{t-1},\;I_{t}\right]+b_{M})\;.$$

where the *W*_*i*_ and *W*_*M*_ represent the trainable weight of the input gate and *M*_*t*_ of the model, respectively. Then the new memory cell of the current time step, *M*_*t*_ can be obtained, which retains a portion of the dependent information from the previous time step:

$$M_{t}=f_{t}\times M_{t-1}+s_{t}\times M_{t}^{\prime }\;,$$

Finally, SMFM can filters the *M*_*t*_ by generating a control factor *o*_*t*_ to obtain the new output *output*_*t*_ of BiLSTM:

$$o_{t}=\sigma (W_{o}\cdot \left[H_{t-1},\;I_{t}\right]+b_{o})$$

$$output_{t}=o_{t}\times tanh\left(M_{t}\right)\;.$$

The loop is repeated and the long-range dependencies of the original semantic features can be learned and aggregated by our SMFM model, resulting in extra significant features and representations of the enhancers.

*2) Ensemble machine learning classifier*: To further boost the performance of enhancer prediction, we established a feature-based ensemble learning classifier to identify DNA enhancers in human cell lines by using fully the interplay between different machine learning algorithms and feature spaces. To demonstrate why we chose these classifiers, we applied different machine learning algorithms to identify DNA enhancers in human cell lines. In a preliminary experiment, we selected the base classifiers from a number of machine learning classifiers including Deep Forest [35], XGBoost [38], LightGBM [39], SVM [34], Random Forest [36], Logistic Regression [40], KNN [41] and GBDT [42]. In particular, we trained the different base classifiers to predict DNA enhancers, and the performance results of the base classifiers are summarized in Table 1. From the results, SVM, Random Forest, and Deep Forest were the top three classifiers in terms of performance, and there is a performance gap between each two classifiers with diversity, which is more suitable for forming the ensemble. Therefore, we finally chose Deep Forest, Random Forest and SVM as the base classifiers of the ensemble classifier. Then, the hard voting scheme is employed to reach the final decision, and it outputs the category with the highest majority of votes in the base classifier:

$$vote=\sum_{i=1}^{n}\frac{BML_{i}\left(v_{t}\right)}{n}\;,$$

where *BML*_*i*_ represents the label generated by the *i*-th base classifier in the ensemble classifier, and then “1” in each generated label indicates that the sample is an enhancer and a 0 indicates that it is not. *v*_*t*_ is the vector that characterizes the *t*-th sequences in dataset. *n* denotes the number of base classifiers in the ensemble model. The classification of the *t*th sequence is judged by the value of *vote*. The *t*th sequence is classified as an enhancer with a *vote* > 0.5, otherwise it is classified as a non-enhancer.

**Table 1:** Results for each base classifier on the training set assessed by four metrics.

Classifier	ACC (%)	MCC	SN (%)	SP (%)
Deep Forest	82	0.651	83.25	81.5
SVM	66.91	0.348	78.56	55.27
Random Forest	69.89	0.408	68.75	70.92
GDBT	66.72	0.338	74.16	59.28
Logistic Regression	65.93	0.327	72.41	59.47
KNN	62.14	0.243	64.65	59.64
LightGBM	66.76	0.339	74.19	59.37
XGBoost	65.46	0.311	70.96	59.97

### D. Parameter settings

The details of the parameter settings for SMFM and the other machine learning algorithms are described below.

*1) Parameters of SMFM*:SMFM contains a number of tunable hyperparameters, which can be specifically divided into the hyperparameters of deep learning-based sequence network and ensemble machine learning classifier. During the optimization of these parameters, we assign the search space for each parameter and explore their optimal combination using 10-fold cross-validation and grid search. After that, the average MCC values (see below) from ten rounds of cross-validation are calculated as the criterion for selecting the parameter combinations. The hyperparameters of SMFM contain mainly the size of the convolution kernel, the number of filters in the convolution layer and the units of BiLSTM. We assign their search spaces as {{1,3}, {3,3}, {3,5}, {5,5}}, {16, 32, 64, 128} and {16, 32, 64, 128}. After optimization, we eventually choose the parameter combinations of 3, 3, 32, 16 and 16, representing the kernel sizes of the first and second convolutional layers, the number of filters in the first and second convolutional layers and the untis of the BiLSTM network, respectively. Indeed, the deep learning-based sequence network is trained using the tensorflow version 2.5.1, and the parameter distribution of each hidden layer in the model adopts the default version of tensorflow. To prevent overfitting, we apply the early stopping method in the training. The hyperparameters of the ensemble machine learning classifier are divided into three components: the first are the hyperparameters of Deep Forest(DF) [35], where we mainly tune the number of estimators in each cascade layer, the number of trees in each estimator, the maximum depth of the cascade forest, decide whether to connect additional predictors at the end, and the type of predictors. Second, we optimize the kernel function in the SVM [34] as well as the values of gamma and C. Third, there are the hyperparameters of random forest [36], which consists of a function measuring the quality of the split and the number of estimators in each tree. Table 2 summarizes the search space of the ensemble learning classifier and the optimal combination for each hyperparameter.

**Table 2:** Best combination of hyperparameters for each classifier.

Base classifier	Search space	Best combination
Deep Forest	‘n_estimators’: {50, 55, 60, 65};	{65, ‘True’, ‘lightgbm’, 30, 25}
	‘use_predictor’: {‘True’, ‘False’};	
	‘predictor’: {‘xgboost’, ‘lightgbm’, ‘forest’};	
	‘max_layers’: {10, 20, 30, 40};	
	‘n_trees’: {20, 25, 30}	
SVM	‘C’: {5, 10, 15, 20};	{5, 1e-3, ‘poly’}
	‘gamma’: {1e-3, 5e-3, 1e-4};	
	‘kernel’: {‘linear’, ‘poly’, ‘rbf’, ‘sigmoid’};	
Random Forest	‘n_estimators’: {60, 65, 70, 75};	{75, ‘gini’}
	‘criterion’: {‘gini’, ‘entropy’}	

*2) Deep learning algorithms*: To elucidate the effectiveness of our proposed model, we compare SMFM with several deep learning models including CNN, RNN and ResNet-1D, a residual network with 1D convolution layers. For CNN, we mainly adjust the number of hidden layers, the number of filters in each layer, the size of the convolution kernel and the learning rate. For RNN, the learning rate and the number of units in hidden layers are selected to optimize RNN. For ResNet-1D, the number of convolution blocks and the activation function are tuned to achieve the best performance. Hyperparameters tuning of these models is performed by grid search.

*3) Machine learning algorithms*: In terms of machine learning algorithms, XGBoost [38], LightGBM [39], SVM [34], Random Forest [36], Logistic Regression [40], KNN [41] and GBDT [42] are employed to compare performance to SMFM. The version of XGBoost [38] is 1.5.1, the version of LightGBM [39] is 3.3.1, and the rest of the model is implemented under the scikit-learn package [43]. In our experiments, we utilize the grid search method to find the optimal parameters for each model.

### E. Evaluation metrics

We use accuracy (ACC), Matthews correlation coefficient (MCC), sensitivity (SN), and specificity (SP) to evaluate the enhancer identification performance of our models.

For DNA enhancer identification, the prediction results can be divided into four categories: true positive (TP), false positive (FP), true negative (TN) and false negative (FN). ACC is the ratio of the number of correctly classified samples to the number of all samples, which most intuitively represents how well a model performs in correctly classifying samples as follows:

$$ACC=\frac{TP+TN}{TP+FP+TN+FN}\;.$$

*SN* is the proportion of true positive samples classified as positive, which characterizes the sensitivity of the model to positive samples.

$$SN=\frac{TP}{TP+FN}\;.$$

As opposed to *SN*, *SP* represents the sensitivity of the model to negative samples, i.e., the proportion of true negative samples among those classified as negative.

$$SP=\frac{TN}{TN+FP}\;.$$

MCC is a metric applied to measure the balanced performance of a binary classification model that considers simultaneously TP, TN, FP and FN to obtain a fair result when an imbalance exists in the dataset:

$$MCC=\frac{TP\times TN-FP\times FN}{\sqrt{\left(TP+FP)\cdot (TP+FN)\cdot (TN+FP)\cdot (TN+FN\right)}}\;.$$

Indeed, MCC characterizes the correlation coefficient between the actual and the predicted classifications, with a value of 1 indicating that the model achieves a perfect performance of the problem, and a value of -1, indicating that the classifier performs even worse than a random prediction.

## Results and discussion

We carried out several experiments to elaborate the effectiveness of our proposed algorithm. At first, we performed ablation experiments on a variety of biological features using the training set to demonstrate the superiority of the features we use. On this basis, we also compared SMFM with some classic deep learning networks and machine learning models. In addition, to elucidate the importance of the dynamic semantic information in the model, we compared the performance of EnhancerBERT models based on different k-mers used to tokenize the enhancer sequences. Moreover, we used an independent test set to compare the performance of SMFM to already existing enhancer prediction models to further investigate the superior performance of SMFM. Finally, we performed motif and interpretability analysis based on the EnhancerBERT attention in SMFM.

### A. Multi-source feature descriptors importance analysis

**Fig 3:**
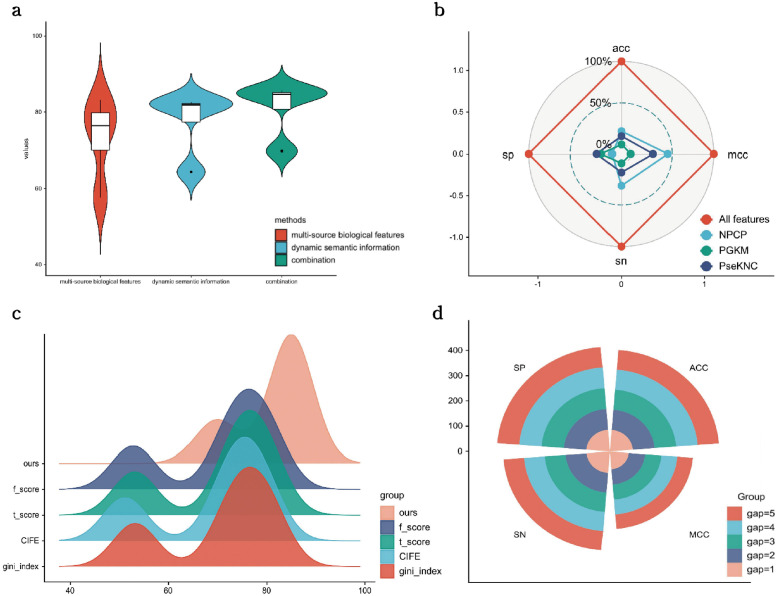
(a) shows the experimental results of ablation of two groups of feature encodings on SMFM, where the fusion of the two feature types achieves best performance; (b) Ablation experiment of multi-source biological features in SMFM, showing percentage of variance of each ablation experiment; (c) illustrates performance of different feature selection methods, where multi-source feature selection can select feature set better than other feature selection methods; (d) compares the specific effects of gap values of PGKM features on the final performance; as the gap value increases, the performance increases.

To begin with, we compared the performance of different types of features including multi-source biological feature-encoding and EnhancerBERT on SMFM, and the results are presented in Fig 3A, where we see that the fusion of the two feature types is better than the individual feature alone. The values of the evaluation metrics of the model after the fusion of the two features were 84.93% ± 0.017, 0.698 ± 0.034, 84.35% ± 0.027, and 85.62% ± 0.026, respectively.

To further verify the effectiveness of the components including Positional gapped k-m-tuple pairs (PGKM), Pseudo K-tuple nucleotide composition (PseKNC), Nucleotide physicochemical properties (NPCP), and their combinations in the multi-source biological feature encoding methods, we performed ablation experiments on them. Specifically, four different experiments were conducted to compare the result of removing each of the four encoding methods from the feature set. The experimental results are shown in Fig 3B. Each individual feature in the figure represents the performance obtained after removing the particular feature. Having all features gets the highest metric values for all four metrics. It is worth mentioning that the stability of the ablated model (with ACC standard deviation value of 0.041) is lower than that of the complete model (with ACC standard deviation value of 0.017) in the cross-validation, indicating that using multi-source biological features, SMFM is able to capture divergent aspects of sequences to support prediction.

In addition, to demonstrate the effectiveness of our proposed multi-source feature selection, we compared our model with different feature selection methods that replace the multi-source feature selection of SMFM to conduct a fair gcomparison. The experimental results are presented in Fig 3C, and confirm that the refined features generated by our method brings a significantly better performance than the other feature selection methods. After training, the features encoded by our method reached an ACC of 84.93% ± 0.017 and MCC of 0.698 ± 0.034, which is about 4.37% and 6.79% higher, respectively than the best performance of any of the other feature selection methods.

In addition, we analysed the effect of different *gap* values in the positional gapped k-m-tuple pairs (PGKM), by testing the performance of the PGKM features with *gap* values of 1,2,3,4 and 5, respectively. The results generated for each gap value are illustrated in Fig 3D that indicates that the performance obtained for *gap* = 5 is optimal since the features with higher *gap* values encapsulate features with lower *gap* values, i.e., features generated for *gap* < 5 are a subset of *gap* = 5, which assists in retaining a portion of the short-distance dependencies in enhancer sequences.

### B. The impact of different natural language processing techniques

**Fig 4:**
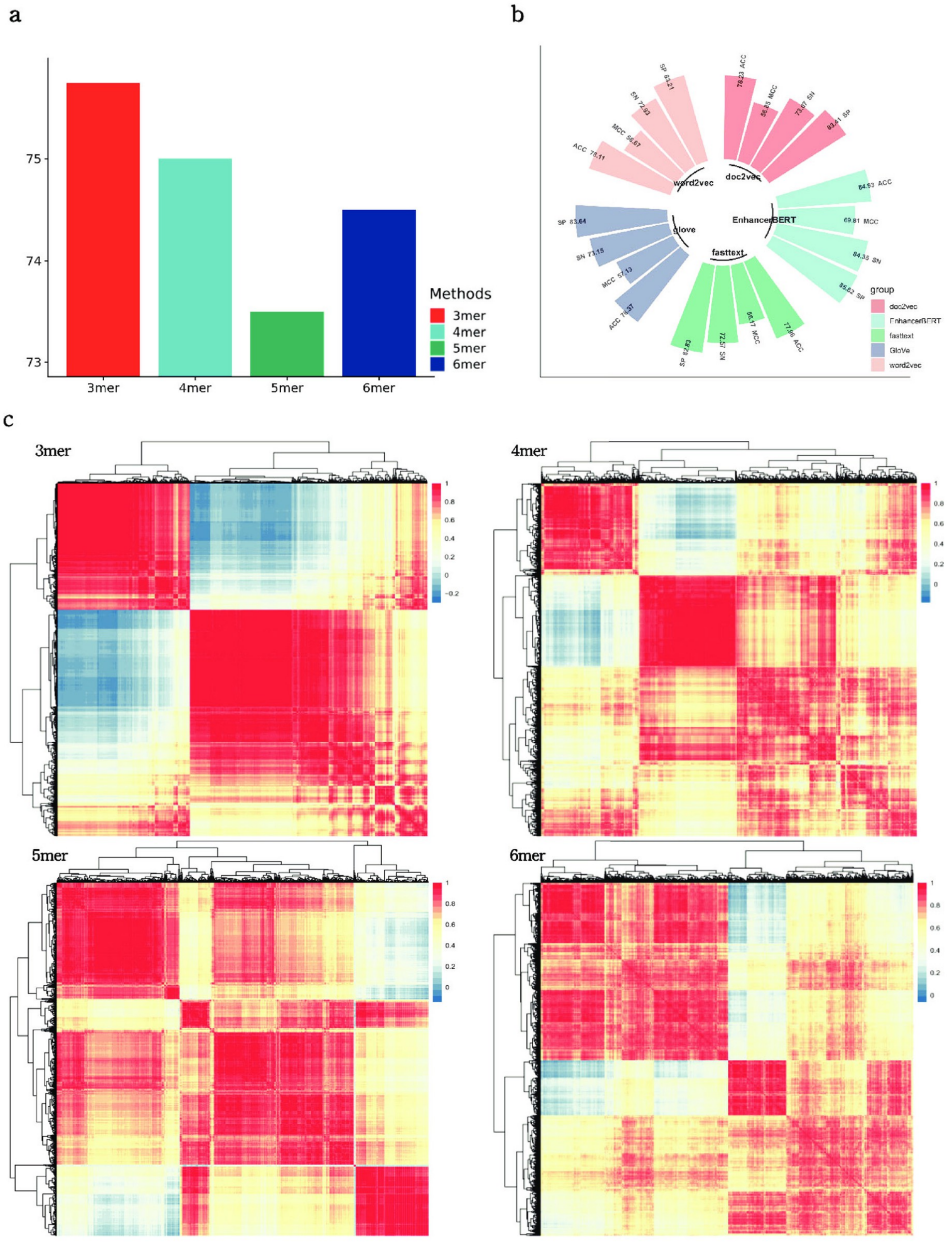
(a) ACC performance of different k-mer EnhancerBERT models, where the 3mer-model achieves the best performance over all models; (b) values of four metrics for assessing performance of EnhancerBERT versus the different NLP technologies, showing that dynamic semantic information in EnhancerBERT has the best characterization capability; (c) compares degree of correlation of different k-mer dynamic semantic information using Pearson correlation coefficient, 3mer-model has the clearest correlationship between features, which support SMFM in identifying enhancers.

To evaluate the effect of *k* values on the identification performance of our model, we tokenized sequences into 3mers, 4mers, 5mers and 6mers to fine-tune different EnhancerBERT models, and separately tested the performance of the dynamic semantic information generated by these different EnhancerBERT models for comparison. The results are summarized in Fig 4A. Through cross validation, we observe that the performance of the EnhancerBERT models are 75.75% ± 0.023, 75.00% ± 0.027, 73.50% ± 0.028, and 74.50% ± 0.021, respectively. To further explore the reason why the 3mer-model achieves the best performance, we calculated the Pearson correlation coefficient between every two features in the k-mer EnhancerBERT and clustered the features based on this to obtain the corresponding correlation heat map. Fig 4C shows the correlation heatmaps with different dynamic semantic information, where it can be observed that 3mers provides the best correlation compared to the other groups, both in terms of degree of correlation and aggregation. From the point of view of performance and correlations, we choose 3mers for the fine-tuning and performed dynamic semantic information extraction of the enhancer sequences.

To investigate the advantage of applying a dynamic semantic information to SMFM, we conducted an experiment comparing EnhancerBERT to existing several static NLP methods, including Word2Vec, FastText, GloVe and Doc2Vec. The results are summarized in Fig 4B. Dynamic semantic information from EnhancerBERT obtains the highest values for all four metrics (ACC of 84.93% ± 0.017 and MCC of 0.698 ± 0.034), well above the metric values of Word2Vec (78.11% ± 0.025 and 0.566 ± 0.046), GloVe (78.37% ± 0.025 and 0.571 ± 0.049), Doc2Vec (78.23% ± 0.026 and 0.568 ± 0.047) and FastText (77.96% ± 0.021 and 0.562 ± 0.056). Further, we compared the performance results of EnhancerBERT with other static NLP methods using the t-test, with *p*-values of 1.9e-2 (Word2Vec), 2.1e-2 (Doc2Vec), 2.2e-2 (GloVe) and 1.8e-2 (Fasttext), respectively, indicating that improvements were significant with EnhancerBERT. We can observe that there is some difference in the sensitivity of the static NLP features to positive and negative samples, and dynamic semantic information can eliminate the difference. Benefit from fine-tune process and multi-head self-attention mechanism, dynamic semantic information contain more relationships about the token position and the dependencies between each nucleotide and its context, resulting in better performance than static NLP technologies. Based on the results, EnhancerBERT model can fully capture the general global contextual characteristics of enhancer sequences.

### C. The Ablation Analysis of the SMFM Model

**Fig 5:**
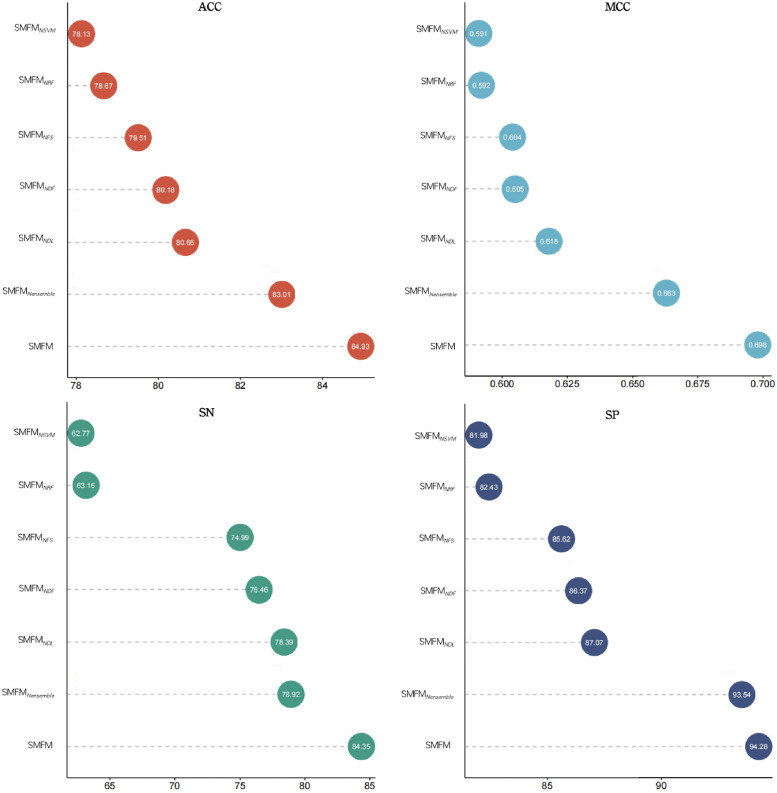
Performance of the different SMFM ablated architectures of SMFM, with ACC, SN and SP values as percent units.

To illustrate the necessity of each module in the SMFM, we performed an ablation analysis for each of its modules. Specifically, we ablated each component of SMFM, including the deep learning-based sequence network fusing CNN and RNN, the stack-based ensemble learning classifier, and each base classifier inside its stack, resulting in the following six scenarios: 1) Remove multi-source feature selection from SMFM and the original multi-source biological features are fed directly into the model, called SMFM_*NFS*_; 2) Remove the ensemble machine learning classifier from SMFM and directly use the deep learning-based sequence network for prediction, called SMFM_*Nensemble*_; 3) Remove the deep learning-based sequence network from SMFM, called SMFM_*NDL*_; 4) Remove SVM from the ensemble machine learning classifier, called SMFM_*NSVM*_; 5) Remove deep forest from the ensemble machine learning classifier, called SMFM_*NDF*_; 6) Remove random forest from ensemble machine learning classifier, called SMFM_*NRF*_. The experimental results are summarized in Fig 5 of assessment by four evaluation metrics. We can observe in Fig 5, that SMFM outperforms all the altered cases (highest ACC value of 84.93% ± 0.017, MCC value of 0.698 ± 0.034, SN value of 84.35% ± 0.027 and SP value of 85.62% ± 0.026). By comparing SMFM_*NFS*_ to SMFM, we see that the feature selection module in SMFM not only improves the prediction performance of the model, but also reduces the number of features of multi-source biological information from 14,891 to 472, thereby significantly reducing the computational time of the model. Comparing SMFM_*NDL*_ to SMFM, we see SMFM shows better performance, also indicating that the deep learning-based sequence network can learn potential features more effectively and capture the implicit relationships and long-distance dependencies, which has a positive impact on the overall performance of the algorithm. Moreover, from the results of SMFM_*Nensemble*_, SMFM_*NSVM*_, SMFM_*NDL*_ and SMFM_*NDF*_, it can be seen that the ensemble of these three machine learning classifiers has a significant impact on the final identification results. In addition, the sensitivity (SN) and specificity (SP) performance analyses in Fig 5 demonstrates that SMFM_*NRF*_ and SMFM_*NDF*_ are comparable; nevertheless, the bias for positive and negative samples is notably different. This phenomenon can be removed when RF, DF and SVM are combined for identification, justifying the combining of these three classifiers. In summary, each module of SMFM is reasonable and valid.

### D. SMFM is superior to other deep learning architectures

**Fig 6:**
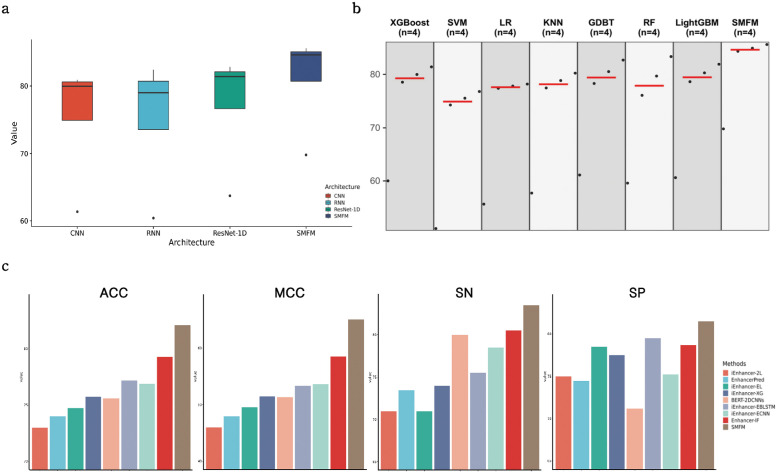
(a) exhibits performance of different deep learning architectures in comparison with SMFM, each box represents four metric values of different architectures; (b) shows performance of different machine learning algorithms in contrast to SMFM, red line shows average value of four metric values of each method; (c) indicating performance of SMFM compared to current state-of-the-art models of enhancer identification on an independent test set, each sub-figure represents comparison result of one of four metrics used in experiments.

To demonstrate the effectiveness of our proposed SMFM, we compared our proposed model with several deep learning architectures including CNN architectures, BiLSTM networks with attention mechanism and ResNet-1D, on the same dataset. Fig 6A displays the results of the different architectures, showing SMFM obtains ACC and MCC values of 84.93% ± 0.017 and 0.698 ± 0.034, respectively, which is the best performance of all four models. For the other models, CNN, BiLSTM and ResNet-1D obtained ACCs of 80.93% ± 0.028, 80.15% ± 0.021 and 81.87% ± 0.016 and MCCs of 0.61 ± 0.056, 0.60 ± 0.044 and 0.64 ± 0.031, respectively, indicating that the learning capability of SMFM is much stronger than a single deep learning model as it synergizes deep learning and machine learning. Moreover, we also note that the results of SMFM are 84.35% ± 0.027 and 85.62% ± 0.026 for SN and SP, respectively, while the results of the other three deep learning models are 80.51% ± 0.075, 77.88% ± 0.024, and 82.82% ± 0.027 for SN and 79.41% ± 0.054, 82.43% ± 0.030, and 80.93% ± 0.042 for SP, revealing that SMFM better addresses the large variability between sequences compared to the other three single deep learning models.

### E. SMFM can provide better performance than several machine learning models

To verify further the effectiveness of SMFM in enhancer identification, we compared our proposed model to seven machine learning models, including XGBoost, LightGBM, SVM, Random Forest (RF), Logistic Regression (LR), KNN, and GBDT. We performed a grid search for each algorithm to achieve the best performance on the dataset, and the detailed information on the tuning parameters of each algorithm can be found in Table 3. As can be seen in Fig 6B, SMFM achieved the best results in all four metrics. SMFM achieved 4.41%, 8.69%, 5.73%, and 2.27% higher values respectively than the other machine learning models for the four metrics. The significant improvement in MCC demonstrates the higher stability of SMFM compared to general machine learning models. Notably, after SMFM, the GDBT classifier obtained better results than the rest of the models, further revealing the effectiveness of ensemble learning in enhancer classification.

**Table 3:** Reference parameters for each of the machine learning algorithms.

ML algorithm	Search space	Best combination
XGBoost	‘max_depth’: {4, 6, 8, 10};	{6, 0.9, 0.5, 0.1}
	‘subsample’: {0.5, 0.7, 0.9, 1.0};	
	‘colsample_bytree’: {0.5, 0.7, 0.9, 1.0};	
	‘learning_rate’: {0.05, 0.1, 0.15, 0.2};	
SVM	‘C’: {5, 10, 15, 20};	{5, 1e-3, ‘poly’}
	‘gamma’: {1e-3, 5e-3, 1e-4};	
	‘kernel’: {‘linear’, ‘poly’, ‘rbf’, ‘sigmoid’};	
LR	‘penalty’: {‘l1’, ‘l2’, ‘elasticnet’, ‘none’};	{‘l2’, ‘liblinear’, 100}
	‘solver’: {‘liblinear’, ‘lbfgs’, ‘sag’, ‘newton-cg’};	
	‘max_iter’: {50, 100, 150, 200};	
KNN	‘weights’: {‘uniform’, ‘distance’};	{‘distance’, 35, ‘euclidean’}
	‘leaf_size’: {25, 30, 35, 40};	
	‘metric’: {‘euclidean’, ‘manhattan’, ‘chebyshev’};	
GDBT	‘n_estimators’: {50, 75, 100, 125}	100, 0.8, 0.6
	‘learning_rate’: {0.2, 0.4, 0.6, 0.8}	
	‘subsample’: {0.5, 0.6, 0.7, 0.8}	
RF	‘n_estimators’: {60, 65, 70, 75}	{60, ‘gini’}
	‘criterion’: {‘gini’, ‘entropy’}	
LightGBM	‘learning_rate’: {0.05, 0.07, 0.09, 0.1}	{0.1, 100, 4, 0.9}
	‘n_estimators’: {50, 75, 100, 125}	
	‘max_depth’: {3, 4, 5, 6}	
	‘subsample’: {0.8, 0.9, 1.0}	

### F. Comparison with existing enhancer identification methods

To further demonstrate the generalization performance and stability of SMFM, we compared SMFM with a number of existing enhancer identification models including iEnhancer-2L [14], EnhancerPred [15], iEnhancer-EL [16], iEnhancer-XG [18], iEnhancer-EBLSTM [22], iEnhancer-ECNN [19], BERT-2DCNNs [24], and Enhancer-IF [21] on an independent test set. iEnhancer-2L [14] is a two-layer classifier built on an SVM model, where the first layer is used to identify whether the sequence is an enhancer and the second layer classifies the strength of the enhancer sequence. EnhancerPred [15] also uses an SVM model to build the corresponding prediction model. iEnhancer-EL [16] applies the ensemble learning idea to obtain a two-layer ensemble classifier. iEnhancer-XG [18] is a two-layer enhancer identification model built using XGBoost [38] and five classical physicochemical features. iEnhancer-EBLSTM [22] and iEnhancer-ECNN [19] bring deep learning to the enhancer identification problem by building ensemble deep learning networks. BERT-2DCNNs [24] construct a 2D CNN network using sequence features extracted from the pre-trained BERT models. Enhancer-IF [21] is an approach for investigating the cell specificity of enhancers using five base classifiers to construct the enhancer identification model on eight different cell lines. The results of the comparative analysis are shown in Fig 6C and Table 4. The performance results of each method on the training set are shown in S1 Table.

**Table 4:** Results of each model on the independent test set using four metrics.

Methods	ACC (%)	MCC	SN (%)	SP (%)
SMFM	**82**	**0.651**	**83.25**	**81.5**
iEnhancer-2L	73	0.460	71	75
EnhancerPred	74	0.480	73.5	74.5
iEnhancer-EL	74.75	0.496	71	78.5
iEnhancer-XG	75.75	0.515	74	77.5
BERT-2DCNNs	75.6	0.514	80	71.2
iEnhancer-EBLSTM	77.2	0.534	75.5	79.5
iEnhancer-ECNN	76.9	0.537	78.5	75.2
Enhancer-IF	79.3	0.585	80.5	78.7

SMFM achieved the highest performance in all four metrics with values of 82% (ACC), 0.651 (MCC), 83.25% (SN) and 81.5% (SP) on the test set, which proves that SMFM has a superior ability to identify DNA enhancers. Compared to BERT-2DCNNs [24], EnhancerBERT in SMFM exhibits a better representation capability. In contrast to several machine learning-based algorithms, SMFM can extract implicit relationships and long-distance dependencies from the original features, which makes the effective information more aggregated. As opposed to the various deep learning-based algorithms, SMFM makes predictions based on ensemble machine learning, which incorporates the diverse perspectives of features. Moreover, it is worth mentioning that the performance results of SMFM on the training and test sets are the closest, while the other methods have a larger gap [14–16, 18], which proves that SMFM is able to maintain some stability between datasets containing different information. Based on all the above, SMFM is better tailored to enhancer identification than the existing methods, and has great potential for exploration of enhancer sequences.

### G. Motif Analysis learned from SMFM

To elucidate the ability of SMFM to extract enhancer motifs, we compared our proposed SMFM with BPNet [44] on this enhancer dataset. Indeed, BPNet is a general and interpretable deep learning model for learning transcription factor (TF) binding motifs in DNA sequences, and then the learned parameters of BPNet are fed into DeepLIFT and TF-MoDISco to detect the motifs. To conduct a fair comparison, similar to BPNet, we also first input the learned parameters of SMFM to DeepLIFT [45] to backtrack signals from the last layer of the two models to calculate the contribution scores of different bases in a sequence to the final identification result, respectively, thus identifying DNA fragments with high contribution scores from the complete sequence. After that, the TF-MoDISco tool [46] was used to scan and cluster the obtained fragments and highlight the significant regions within the sequences by the feature importance scores, and motifs are then aggregated by aligning fragments from each cluster. On this basis, we finally identified 56 motifs with widths ranging from 15 to 62 for SMFM while 47 motifs with widths ranging 11 to 69 for BPNet.

To further verify the validity of the motifs captured by the two algorithms, we extracted the corresponding position weight matrices (PWM) from fragments clusters identified by SMFM and BPNet, respectively and visualized the motifs according to the sequence background of enhancer dataset (0.284 for A and T and 0.216 for C and G). Then, we input the PWMs of the two sets of motifs obtained by SMFM and BPNet into the TOMTOM algorithm [47] separately for comparison with experimentally verified motifs in the transcription factor motif database, JASPAR CORE [48] with a significant E-value threshold of 0.05. S2 Table. summarized the comparison of the meaningful motifs detected by SMFM and BPNet, SMFM finally obtained 45 sets of comparison results corresponding to 28 motifs with different IDs in the database, while BPNet obtained 28 sets corresponding to 17 motifs with different IDs. From the table, we observe that the meaningful motifs obtained by SMFM captured the majority of the motifs obtained by BPNet. Moreover, SMFM is able to detect more normal and reverse complementary motifs compared to BPNet (e.g., MA1274.1, MA1403.1, MA0528.1, MA0538.1, etc.). In summary, BPNet is a motif detection tool for a wide range of gene sequences, while SMFM integrates dynamic semantic information for enhancer sequences and multi-source biological properties, thus providing a more comprehensive performance for detecting motifs in enhancers than BPNet.

For easy reference, we put the results of the comparison of the motifs obtained from SMFM and BPNet with those from the JASPAR database in S3 and S4 Tables, respectively, which are also available in the SMFM web server http://39.104.69.176:5010/. In addition, the codes of different computational algorithms for detecting motifs are available at https://github.com/no-banana/SMFM-master.

### H. Interpretability analysis of SMFM

**Fig 7:**
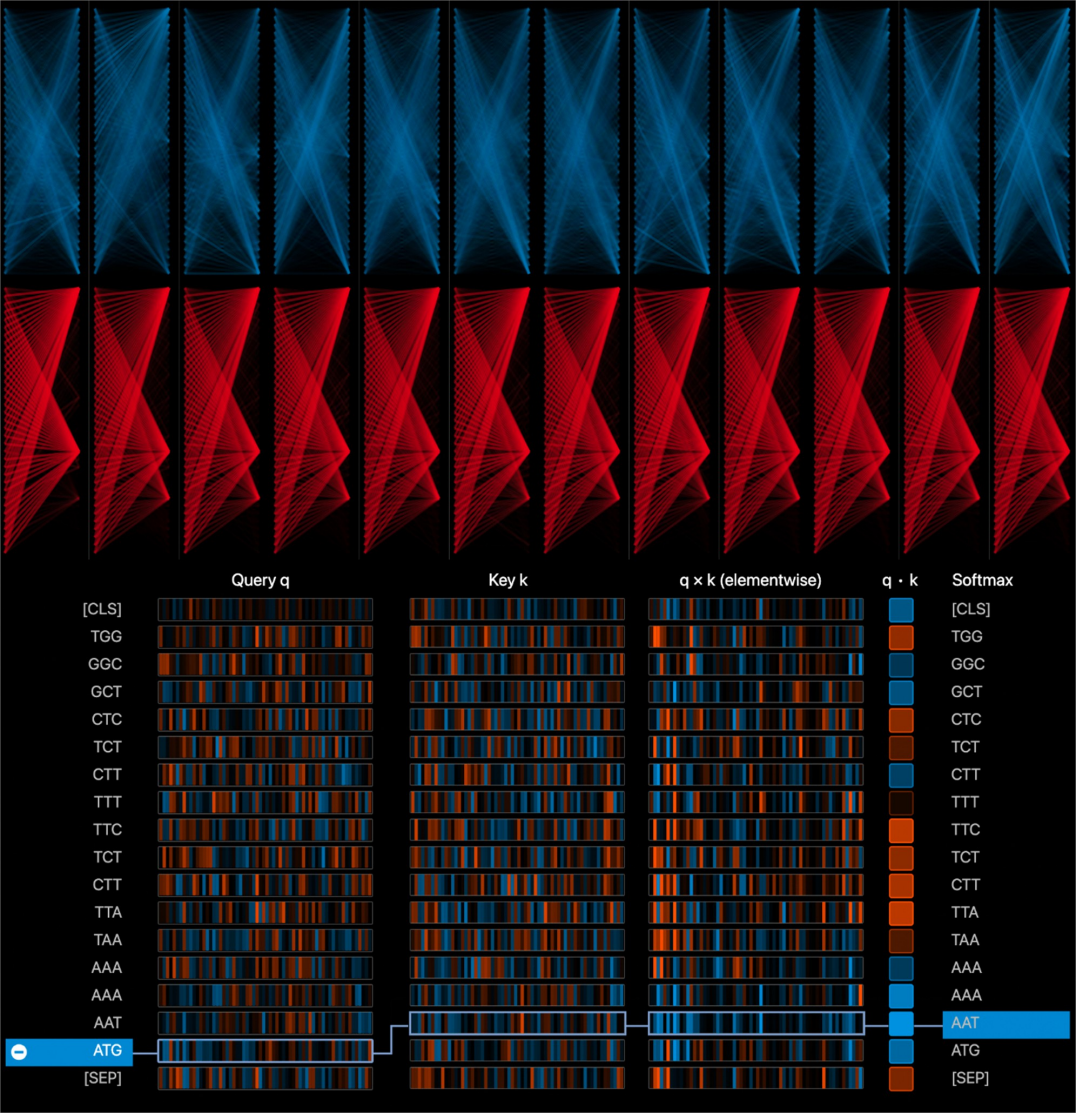
Top half shows a bird’s eye view of the attention distribution of the different attention heads in two different layers of EnhancerBERT, the columns represent attention heads of each EnhancerBERT layer, and rows represent layers of EnhancerBERT. With iterating of the EnhancerBERT layer, the attention scores of each attention head gradually concentrate in some key regions of input enhancer sequence; Bottom half visualizes the process of attention score calculation, where first and second columns represent *Query* vector and *Key* vector, respectively. The framed up vectors show the two most relevant tokens in the enhancer sequence.

**Fig 8:**
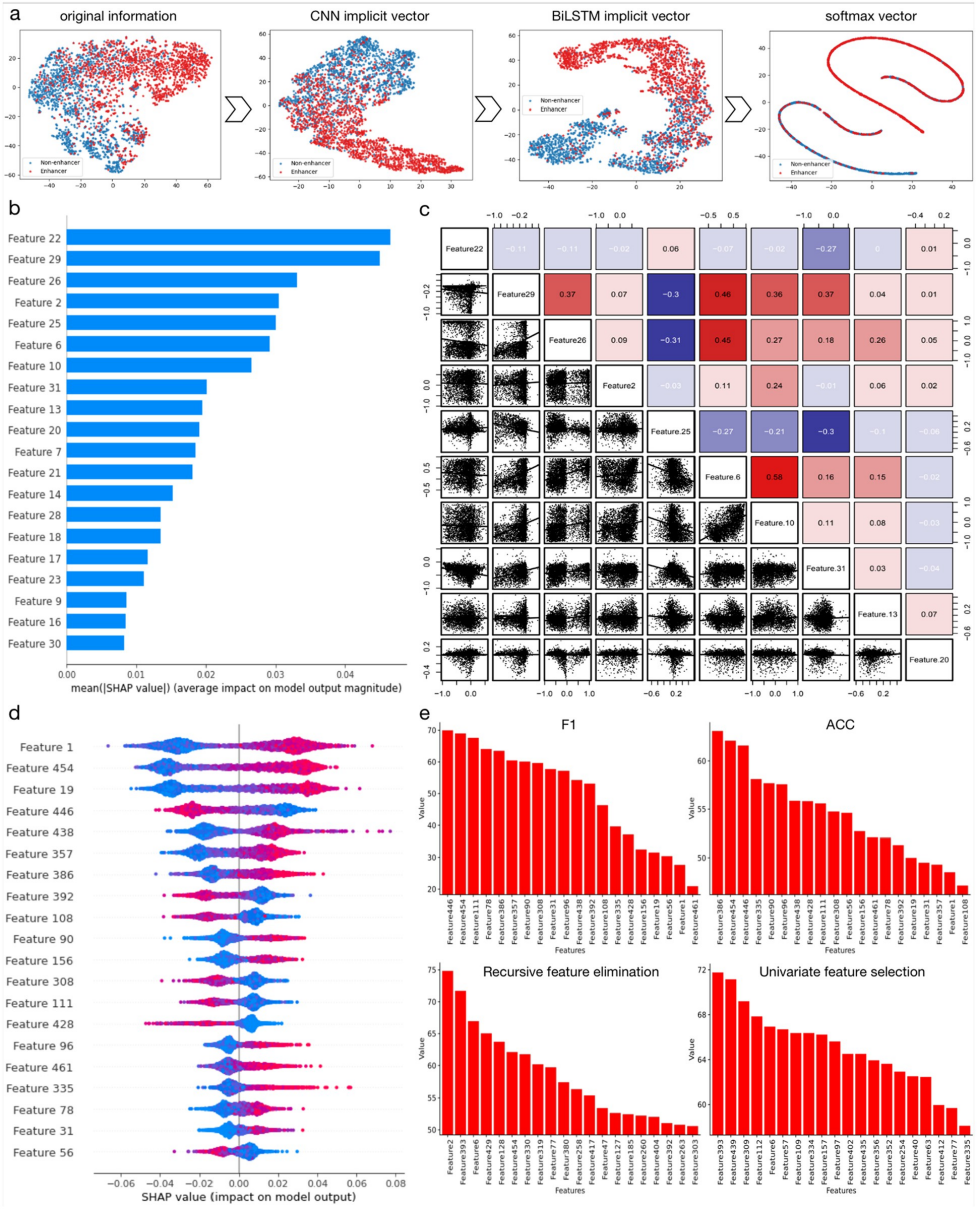
(a) shows the t-SNE results of deep learning-based sequence network for different hidden layers of the dynamic implicit relation and long-distance dependency process in dynamic contextual features; (b) The top 20 features of dynamic semantic information, the higher the SHAP value, the greater the influence of the feature in the classification; (c) Correlations among the top 20 features of dynamic semantic information; (d) The top 20 features of the multi-source biological features having the highest impact on classification; (e) Feature rankings for enhancer identification, where the two sub-figures above are ranked based on F1 and ACC metrics, both rankings are measured based on random forest classifier building under scikit-learn package. The two sub-figures at the bottom are ranked using RFE and UFS feature selection methods.

To verify the effectiveness of extracting dynamic semantic information from EnhancerBERT, as shown in Figs 7 and 8, we explored different aspects of attention weights in EnhancerBERT. In the top half of Fig 7, we provide all the attention heads corresponding to a given sequence in the first attention layer (shown in blue) and the fourth attention layer (shown in red) of EnhancerBERT. It can be seen that after two iterations of the layers, the attention scores of the attention heads in each layer gradually accumulate in some key regions of the sequence that have a large influence on the identification decision.

The bottom half of Fig 7 demonstrates how the attention head of each layer of EnhancerBERT generated the corresponding attention scores for a given sequence; where *Query q* and *Key k* represent the *Query* vector and *Key* vector in the model. Based on these, the attention scores between different tokens can be calculated according to the formula described in the EnhancerBERT section. In this figure, the positive values are displayed in blue, with higher values becoming darker, while negative values are displayed in orange, with lower values becoming darker. Here, we choose the attention scores of the sequence token ‘ATG’ calculated by the first attention head of the last layer of EnhancerBERT as an example, and observe that the attention values between token ‘ATG’ and other tokens in the selected attention head do not decay noticeably with increasing distance, indicating that EnhancerBERT preserves the long-distance dependence information and short-distance information in the sequence successfully.

In addition, we explored the contribution of refined multi-source biological features and implicit dynamic semantic information to enhancer identification. The analysis results are shown in Fig 8. To better explain the learning process of dynamic semantic information in SMFM, we extracted the output of each hidden layer in the deep learning-based sequence network during the training process and projected each hidden vector onto a two-dimensional view using t-SNE. As shown in Fig 8A, the first subplot represents the t-SNE results of the original dynamic semantic information that can be understood as the entire sample points not showing any representative clusters. The second subplot displays the t-SNE result after two layers of CNN processing in the deep learning-based sequence network, where the hidden vectors have a regular distribution. The third subplot reveals the t-SNE results after processing by BiLSTM, where we observed a more obvious clustering distribution, indicating that the implicit relationships and information between features have been adequately captured. Finally, we feed the implicit vector into a softmax classifier and yielded the fourth t-SNE subplot with clear classification results. Fig 8B reflects the impact of each feature of the top 20 features of the implicit dynamic semantic information on identification of different DNA enhancer sequences, where higher SHAP values indicate that the particular feature plays a more positive role in the final prediction decision. Fig 8C shows relationships between the top 20 features, where red indicates positive correlation between the features of the row and column, while purple indicates negative correlation. It can be seen that after learning of deep learning-based sequence network in SMFM, the correlation between features is further amplified; for instance, feature 6 has significant positive correlation with features 29, 26 and 10, which indicates these features are synergistic. Similar conclusions can be drawn between features 29, 26 and 31. Fig 8D reflects the influence of each feature of the top refined biological features, where red color indicates a positive effect: the higher the feature value of the feature, the more likely the sequence is predicted to be an enhancer, and the blue color indicates a negative effect: where the higher the feature value, the more likely the sequence is predicted to be a non-enhancer. We see that different features may have various contributions to final output. Therefore, for the best characterization of enhancer sequences it is better to fuse features together. In the next step, we performed single feature ranking analysis on the top 20 features of the refined multi-source biological features by using the random forest model. Fig 8E summarizes the results, where the two histograms at the top show features of the top 20 SHAP value ranking based on ACC and F1 metrics, respectively. Besides, to obtain the ranking of feature importance for different views, we ranked the feature importance of the refined multi-source biological features using recursive feature elimination [49] and univariate feature selection [50] (as shown in the two histograms at the bottom of Fig 8E). It can be seen that the feature ranking based on SHAP values is totally different from feature ranking based on metrics and feature importance, indicating that there is a large differential expression when characterizing enhancer sequences using only the physicochemical and sequential features. This, on the other hand, reflects the necessity and validity of extracting dynamic semantic information from EnhancerBERT to alleviate this differential expression.

### I. SMFM enables efficient characterization of placental-specific enhancers

**Fig 9:**
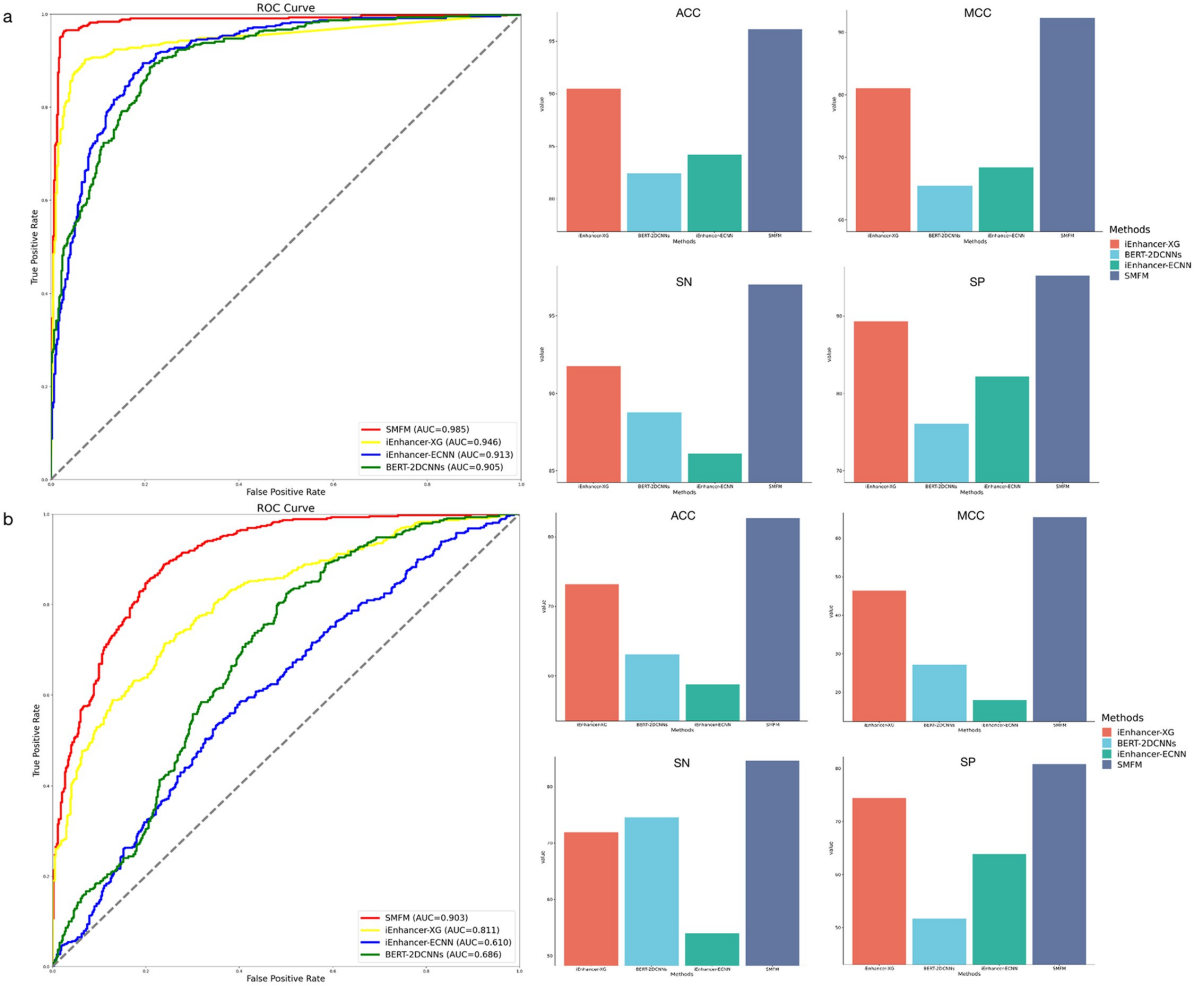
(a) Performance of first step of different enhancer identifying methods compared to SMFM, where the left sub-figure illustrates the AUC performance of SMFM, iEnhancer-XG, iEnhancerECNN and BERT-2DCNNs; (b) shows performance of the second step experiment using different methods.

The placenta is an essential organ for a successful pregnancy and has a variety of basic functions, including the delivery of nutrients to the developing fetus and the protection of the fetus from infectious diseases [51]. Research also indicates that placenta dysfunction is related to pregnancy complications—preeclampsia and preterm birth (PTB), etc [52–55]. Precise control of gene expression is critical for fetal development during pregnancy, and gene regulatory enhancers play a mediating role in controlling gene expression and contribute significantly to development and disease [56–58]. Therefore, the identification of active enhancers in placental tissue is extremely crucial. Here we designed an experiment for 4,562 placental enhancers [59] and then compared the experimental results of SMFM with other existing enhancer methods. To conduct a fair experiment in the placental enhancers task, we did not perform targeted parameter tuning for all methods used for comparison. We directly used the best hyperparameters of each method obtained from the previous experimental analysis, which can better illustrate the robustness of our algorithm. In our study, we first use 4,562 non-enhancers as negative samples, and utilized different methods to identify placental enhancers. In a second step, we replaced negative samples with the same number of enhancers from the human embryonic kidney cell line (HEK293) to test the ability of different methods to distinguish enhancers in placental tissue. We tested the performance of SMFM, iEnhancer-XG [18], iEnhancer-ECNN [19], and BERT-2DCNNs [24] in this experiment, and the experimental results are summarized in Fig 9. In the first experiment, SMFM identified enhancers very well and achieves the highest values for the five metrics, which are 0.985 of AUC, 0.962 of ACC, 0.923 of MCC, 0.97 of SN and 0.953 of SP. In the second experiment, SMFM also showed strong performance in distinguishing enhancers from different tissues with 0.903 of AUC, 0.827 of ACC, 0.655 of MCC, 0.846 of SN and 0.808 of SP. Although the performance of each method decreased in the task of distinguishing placental enhancers from those in other tissues, SMFM remained the most stable and highest performing method.

**Fig 10:**
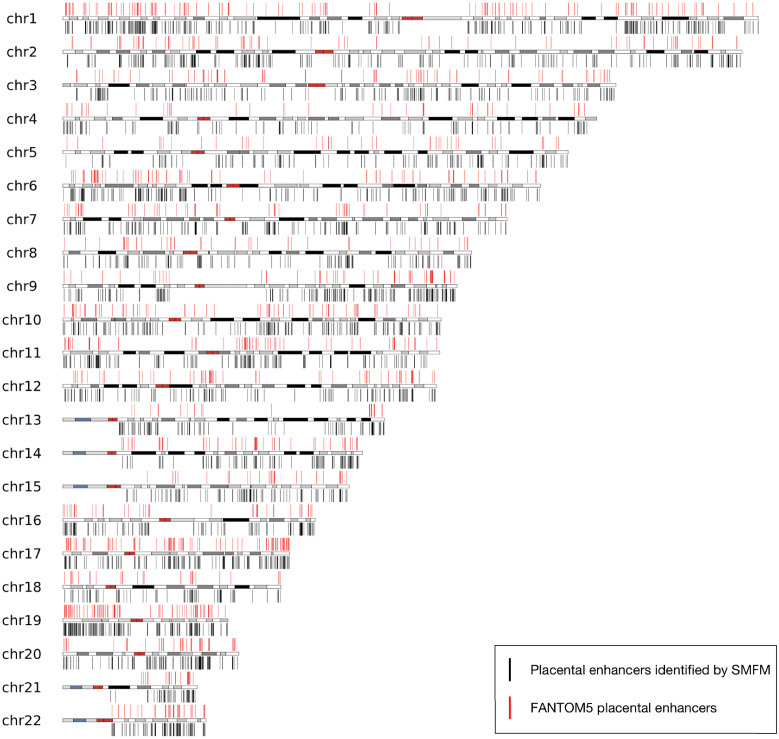
Visualization of placental enhancers identified by SMFM and FANTOM5 placental enhancers on hg19 autosomes, where red lines indicates placental enhancers from FANTOM5 atlas, the identified placental enhancers are shown using black lines. The red regions in autosomes are centromeres, and white regions and regions colored from gray to black represents Giemsa negative and positive regions, respectively. The highly variable and tightly constricted regions on the p-arms of 13, 14, 15, 21, 22 chromosomes that cannot be predicted are shown as blue and gray.

After obtaining results of SMFM in the first experiment, we visualized the samples classified as placental enhancer by SMFM on 22 human autosomes and compared them with the distribution of known placental enhancers on these chromosomes from the FANTOM5 atlas [60]. The visualization and comparison results are demonstrated in Fig 10. It can be observed that the distribution of positive samples obtained by SMFM (shown in black) is generally consistent with the distribution of placental enhancers on chromosomes in the FANTOM5 atlas (shown in red), indicating SMFM brings accurate and efficient characterization of placental enhancers.

We then carried out several experiments to validate the relevance of the placental enhancers identified by SMFM from a gene regulation perspective. We first conducted the enrichment analysis including gene ontology (GO) and kyoto encyclopedia of genes and genomes (KEGG) enrichments for genes that are regulated by the placental enhancers identified by SMFM.

**Fig 11:**
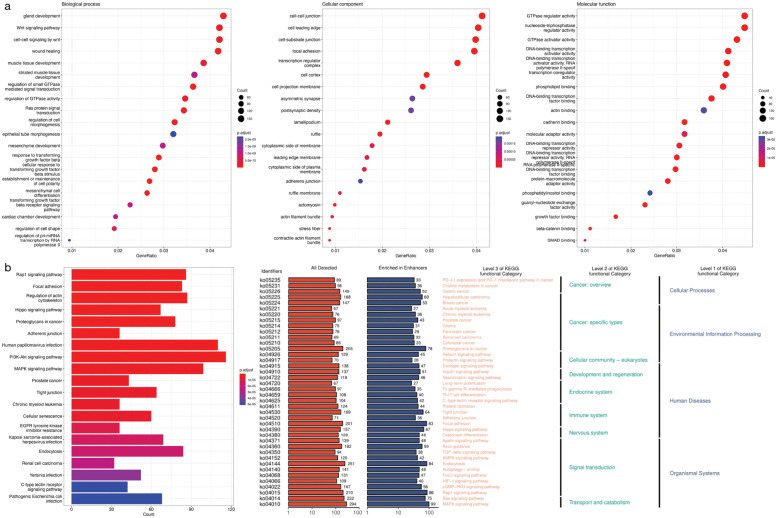
Genomic enrichment analysis of placental enhancers identified by SMFM. (a) The top 20 categories of gene ontology (GO) analysis ordered by *p*-values, including biological process, cellular component, molecular function. (b) shows the top 20 KEGG enrichment pathways ordered by *p*-values and KEGG classified by different functional category levels of KEGG.

Fig 11A shows the top 20 types of GO enrichment ordered by *p*-values. It is worth noting that the top five enriched biological processes of GO are gland development (GO:0048732), wnt signaling pathway (GO:0016055), cell-cell signaling by wnt (GO:0198738), wound healing (GO:0042060) and muscle tissue development (GO:0060537). It can be seen that the majority of enriched biological processes are associated with various tissue development pathways, therefore highly related to development of placenta, successful pregnancy and embryonic development [61–64]. In addition, the top five enriched cellular components are cell-cell junction (GO:0005911), cell leading edge (GO:0031252), cell-substrate junction (GO:0030055), focal adhesion (GO:0005925) and transcription regulator complex (GO:0005667). The top five enriched molecular functions are GTPase regulator activity (GO:0030695), nucleoside-triphosphatase regulator activity (GO:0060589), GTPase activator activity (GO:0005096), DNA-binding transcription activator activity (GO:0001228) and RNA polymerase II-specific DNA-binding transcription factor binding (GO:0061629). In addition, the result of the KEGG enrichment analysis is summarized in Fig 11B. The left sub-figure displays the top 20 of KEGG enrichments ordered by *p*-values. The pathways can also be annotated and classified as functional categories of KEGG at three different levels, as shown in the right sub-figure, where we learn that the pathways can be divided into four categories for level one, including cellular processes, environmental information processing, human diseases and organismal systems, and different functional categories for level two. Among the pathways, most are critical for early embryonic development. For example, Rap1 signaling pathway, which controls important processes such as cell adhesion, cell-cell junction formation and cell polarity. In addition, the regulation of actin cytoskeleton is responsible for regulating the formation of new individuals from embryonic cells [64, 65]. Based on the above analysis, we can conclude that the genes regulated by the placental enhancers identified by SMFM are highly associated with embryonic development and successful pregnancy, which further validates the effectiveness of SMFM for discerning placental enhancers.

### J. SMFM outperforms existing methods on a large-scale dataset

To further validate the predictive ability of SMFM, we investigated its ability on a large-scale dataset from the candidate cis-Regulatory elements (cCREs) in BENGI [66]. To construct this dataset, we collected 30,000 enhancer-like sequences from human cCREs that were longer than 400 bp but shorter than 600 bp and truncated them to 400 bp. Then, we excluded the homologous sequences using the CD-HIT tool with a sequence similarity threshold of 60%. Finally, we obtained 26160 sequences as positive samples in our dataset. Of note, due to the shortage of publicly available high-confidence datasets of non-enhancer sequences, inspired by Dao et al [67], we sampled each pair of human cCREs more than 400 bp apart as negative samples. Then, we removed homologous sequences that shared >60% of their bases with other non-redundant negative and positive samples. Finally, the dataset contained 26160 positive samples and 26160 negative samples with a sample length of 400bp.

**Fig 12:**
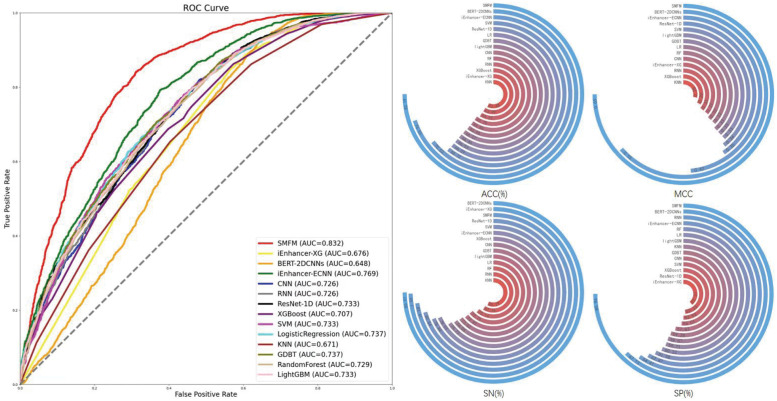
Performance of other enhancer identification methods compared to SMFM on a large-scale dataset, where the left panel illustrates the AUC performance of SMFM, baseline methods, classical deep learning networks, and multiple machine learning classifiers. The right panel shows the four performance measure metrics (ACC, MCC, SN and SP) for each methods.

For comparison on this large dataset, we compared the SMFM algorithm with other baseline methods, including iEnhancer-XG, iEnhancer-ECNN, BERT-2DCNNs, several deep learning and machine learning methods on this dataset. Note that we did not tune the hyperparameters of each method for this dataset in order to better validate the robustness of each method. The experimental results are summarized in Fig 12. It can be seen that SMFM achieved the best performance of all the methods on this large-scale dataset (0.808 for AUC, 0.822 for ACC, 0.655 for MCC, 0.834 for SN, and 0.810 for SP), indicating that SMFM has stronger generalization ability compared with other comparative methods. Moreover, in terms of sensitivity and specificity, SMFM is more balanced for identifying positive and negative samples in the dataset, while the rest of the methods are biased towards the classification of positive samples. In addition, we observed that the method ranking second was iEnhancer-ECNN [19], a method that also uses ensemble learning, indicating that ensemble machine learning classifiers are more accurate at predicting regulatory DNA enhancer sequences. In summary, the experiment also validates the powerful predictive ability of our model.

### K. The SMFM web server

To facilitate use by researchers, we developed a web server for SMFM that allows identifying whether a sequence is an enhancer or not, and this prediction webserver link is available at http://39.104.69.176:5010/. The web server guides users in generating dynamic semantic information and multi-source biological features corresponding to their dataset, and then the user receives the user-generated files to make predictions on the dataset. In addition, the successfully submitted jobs and prediction results are sent to the contact address of the users, including the results of each base classifier and the final results using SMFM. Furthermore, we provide the datasets used in this study, including the training set and independent test set, which can be downloaded directly from the web server. Finally, if users are interested, they can also download the corresponding original EnhancerBERT models from the web server.

## Discussion

In this study, we propose SMFM, a novel method for identifying and characterizing DNA enhancers using a stacked multivariate fusion model. To gather all the useful information from enhancer sequences, the multi-source biological features and dynamic semantic information are extracted and fused to construct feature schemes with excellent representation. After that, a deep learning-based sequence network synergized by CNN and BiLSTM networks is proposed to retrieve the implicit relations and long-distance dependencies. Then, an ensemble machine learning classifier was developed for training based on the refined multi-source features and dynamic implicit relations obtained from the deep learning-based sequence network to predict DNA enhancers in human cell lines. We evaluated SMFM on a benchmark set including 1484 enhancers and 1484 non-enhancers, and then demonstrated the advantages of SMFM over existing methods on an independent test set. In addition, by conducting motif and interpretable analyses, we explain what SMFM has learned to achieve better performance, while revealing how SMFM focus to key functional fragments of the enhancer sequences. Meanwhile, we also designed an experiment to explore characterization ability of SMFM for tissue-specific enhancers, and the analysis indicated that placental enhancers identified by SMFM are effectively associated with embryo development and normal placental functions such as nutrient transport.

However, there is still much room to improve. For example, the current graph neural network achieved remarkable results in multiple fields, and we will attempt to model the DNA sequence structure based on the obtained graph data. On the other hand, it will be interesting to define the notions between language model and enhancer sequences to provide more biological interpretability, subject to data availability in the future.

## Supporting information

S1 Table Results of each model on the training set measured by four metrics. (XLSX)

S2 Table Visualization of meaningful motifs detected by SMFM and BPNet. (XLSX)

S3 Table Visualization of 56 motifs detected by SMFM. (XLSX)

S4 Table Visualization of 47 motifs detected by BPNet. (XLSX)
